# Parity improves anti-tumor immunity in breast cancer patients

**DOI:** 10.18632/oncotarget.20756

**Published:** 2017-09-08

**Authors:** Anna-Lena Krause, Florian Schuetz, Marc Boudewijns, Maria Pritsch, Markus Wallwiener, Michael Golatta, Joachim Rom, Joerg Heil, Christof Sohn, Andreas Schneeweiss, Philipp Beckhove, Christoph Domschke

**Affiliations:** ^1^ Translational Immunology Division, German Cancer Research Center (DKFZ), Heidelberg, Germany; ^2^ Department of Gynecology and Obstetrics, Heidelberg University Hospital, National Center for Tumor Diseases (NCT), Heidelberg, Germany; ^3^ Regensburg Center for Interventional Immunology (RCI) and University Medical Center of Regensburg, Regensburg, Germany

**Keywords:** pregnancy, breast cancer, anti-tumor T cells, regulatory T cells, tumor-associated antigens

## Abstract

Compared to nulliparous women, parous women have an up to 50% lower lifetime risk of developing breast cancer. An endogenous mechanism to prevent the development of cancer is the destruction of tumor cells by T cells that recognize tumor-associated antigens (TAA). Since a number of TAA are also highly present in the breast and placenta of pregnant women, we investigated the induction and characteristics of spontaneous T cell responses against TAA during pregnancy.

To this end, we collected peripheral blood from healthy nulliparous, primigravid and parous women, as well as from breast cancer patients. IFN-γ ELISpot assays were performed to measure the intensity and specificity of T cell responses against 11 different TAA. The impact of TAA-specific Treg cells on anti-TAA responses was assessed by performing the assay before and after depletion of CD4^+^CD25^+^ T cells. The antigenic specificities of these Treg cells were analyzed by the Treg specificity assay. Furthermore, we conducted flow cytometric analyses to determine the memory phenotype and cytokine secretion profile of TAA-specific T cells.

Our results demonstrate that pregnancy induces functional and long-lived memory and effector T cells that react against multiple TAA. These persist for many decades in parous females, but are not found in age-matched females without children. We also detected TAA-specific Treg cells, which suppressed strong effector T cell responses after delivery. Nulliparous breast cancer patients displayed median TAA-specific effector T cell responses to be decreased threefold compared to parous patients, which could be restored *in vitro* after depletion of Treg cells.

## INTRODUCTION

Parity is associated with a 50% decreased lifetime risk of developing breast cancer [1]. However, the mechanisms behind the protective effect remain to be revealed. Since T cell immunity plays a major role in breast cancer prognosis, we here studied a potential influence of parity on breast tumor-specific T cell responses.

The hypothesis that lymphocytes can recognize and eliminate continuously-arising transformed cells was established during the late 1950s by Burnet and Thomas. To date, accumulating evidence supports this concept of “cancer immunosurveillance”: Anti-cancer immune responses are initiated in many breast-, and other cancer patients *upon* recognition of tumor-associated antigens (TAA) by T lymphocytes [2, 3]. Both the presence of TAA-specific T lymphocytes in the blood [4, 5] and the accumulation of effector and memory T cells [6] correlate with an improved survival of breast cancer patients. Thus, the strength of endogenous tumor-specific effector/memory T cell responses determines the outcome of patients with breast cancer.

On the other hand, immune suppressive cells, such as CD4^+^ CD25^+^ regulatory T lymphocytes (Treg), can promote tumor growth. Treg cells play a major role in maintaining self-tolerance, but can also suppress the anti-tumor activity of TAA-specific effector T lymphocytes. Therefore, the intratumoral accumulation of Treg cells is associated with increased tumor grade, lymph node involvement, reduced overall survival and increased risk of relapse in breast cancer patients [7]. Accordingly, *in vivo* reduction of Treg cells with low-dose cyclophosphamide was shown to induce tumor-specific T cell responses in breast cancer patients, which correlated with improved survival [8].

Breast carcinomas overexpress a broad range of TAA which can be recognized by endogenous effector and regulatory T cells [9–11], including carcinoembryonic antigen (CEA), the melanoma-associated antigen (MAGE)-A3, mucin (MUC)-1, the human epidermal growth factor receptors EGFR and HER2, mammaglobin A and heparanase (HPA). Overexpression of TAA is already initiated in ductal carcinoma *in situ*, the most common preinvasive lesion of the breast [12]. Interestingly, many TAA are also associated with pregnancy. An increased expression of multiple TAAs was demonstrated, e.g., in the lactating breast, placenta and blood serum of pregnant women [13–15], and spontaneous effector T cell responses against one of these antigens, MUC-1, were observed in pregnant women [16]. Thus, it is conceivable that pregnancy might induce a population of endogenous breast tumor-specific memory T cells that could confer anti-tumor immunity to breast cancer patients. On the other hand, pregnancy is associated with an increased activity of immune tolerance mechanisms, including the induction of Treg cells, which prevent the mother's immune system to react against the embryo [17]. Therefore, the establishment of TAA-specific memory T cells in pregnant women might be inhibited or counterweighed by the induction of TAA–reactive regulatory T cells during pregnancy or breast cancer development.

We here studied whether pregnancy results in the formation of long-lasting – protective, or inhibitory – T cell populations specific for breast cancer-associated antigens and whether such pregnancy-induced T cell responses do influence T cell immunity during breast cancer. We show that pregnancy induces long-lived memory and regulatory T cell responses against multiple breast cancer-associated antigens that persist for many decades, but are not found in age-matched females without children. Previous pregnancies thereby result in a median threefold increase of tumor antigen-specific effector T cells in parous compared to nulliparous breast cancer patients. In addition, tumor-specific T effector cell responses were strongly suppressed by TAA-reactive Treg in nulliparous women, while the presence of TAA-reactive Treg cells had only a moderate impact on TAA-reactive Teff cell responses in parous breast cancer patients. Taken together, our findings demonstrate that pregnancy results in a TAA-specific T cell memory, which lasts lifelong and which might improve anti-tumor immunity throughout breast cancer disease.

## RESULTS

### Parous women harbor functionally active memory T cells reactive against multiple breast tumor-associated antigens

**Figure 1:**
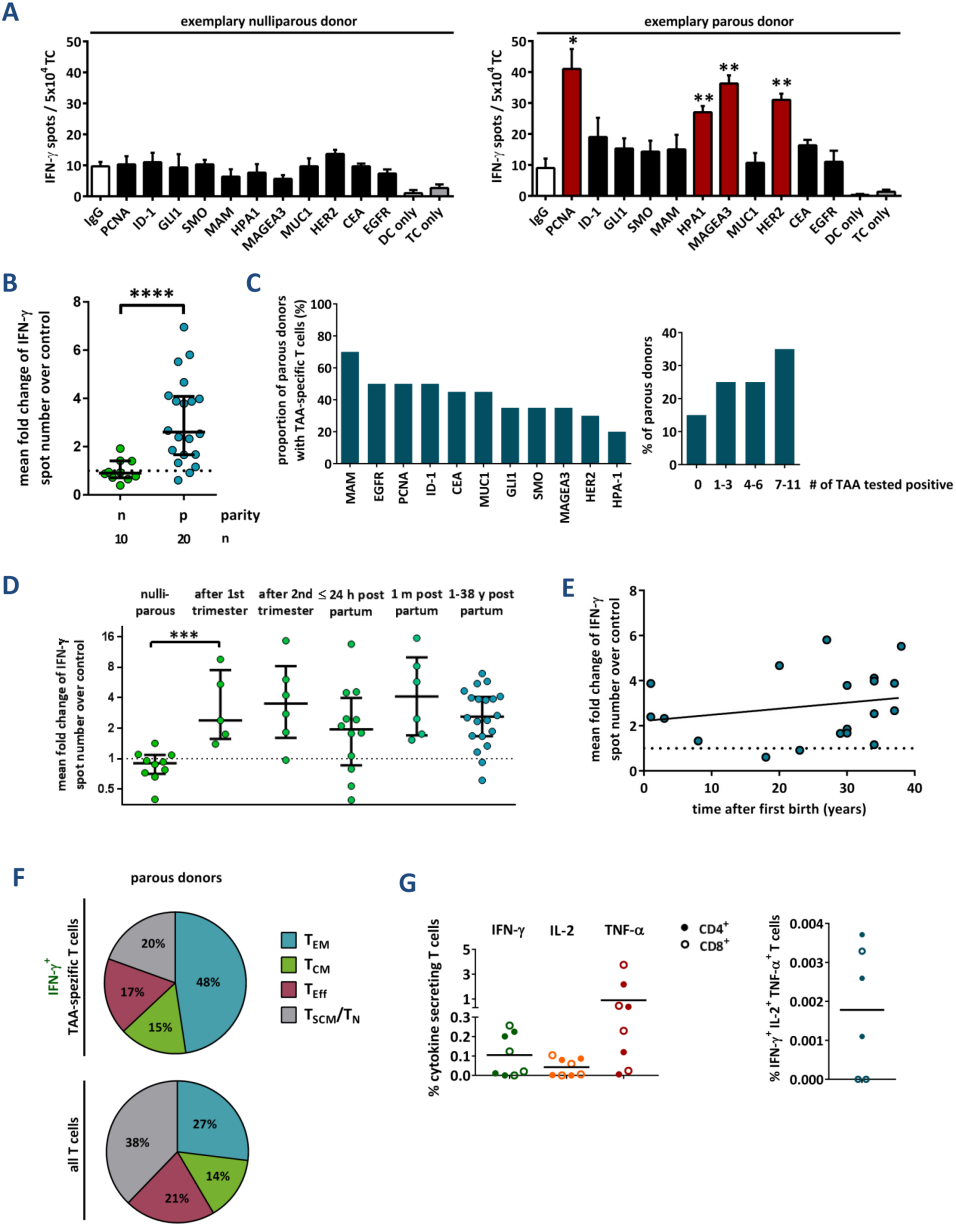
Tumor-associated antigen-reactive T cells are induced during pregnancy and persist for decades **(A)** Primary data of two exemplary IFN-γ ELISpot assays. Peripheral blood T cells of a healthy nulliparous and a healthy parous woman were stimulated with a (potential) TAA, the negative control antigen human IgG, or without antigen (DC, dendritic cells; TC, T cells), respectively. Data represent mean spot numbers of 3 wells per antigen ± SEM. A T cell response was defined positive when spot numbers in test wells were at least 2-fold higher and significantly (p ≤ 0.05) higher compared to spot numbers of negative control antigen as determined with the unpaired 2-tailed t test. ^*^ p ≤ 0.05, ^**^ p ≤ 0.01. **(B-D)** Cumulative results of IFN-γ ELISpot assays. Data points depict the mean fold change of test antigens over negative control per healthy donor (with median ± interquartile range). N: nullipara, p: para. P values were calculated using the unpaired 2-tailed t test comparing the pooled mean log fold change of test antigens over negative control. ^***^ p ≤ 0.001, ^****^ p ≤ 0.0001. (B) TAA-reactivity of peripheral T cells from nulliparous vs. parous women. (C) Proportion of parous females tested positive for single antigens (left) and number of antigens tested positive per donor (right). (D) Peripheral blood was also taken from primiparous women during pregnancy at weeks 14 or 21, and within the first 24 h as well as 1 month after delivery. **(E)** TAA-reactivity of peripheral T cells from parous women in relation to the time after the donor's first delivery. Line: linear regression. **(F)** Memory phenotype of TAA-specific IFN-γ secreting and total T cells, respectively, from four parous women. Mean percentage of naïve and stem cell-like memory T cells (T_N_, TSCM, both CCR7^+^ CD45RO^−^), central memory T cells (T_CM_, CCR7^+^ CD45RO^+^), effector memory T cells (T_EM_, CCR7^−^ CD45RO^+^), and terminally differentiated effector T cells (T_E_, CCR7^−^ CD45RO^−^). **(G)** Cumulative results of cytokine secretion assays with T cells from healthy parous donors. Total T cells were stimulated with a TAA mixture or human IgG as a control. CD4^+^ and CD8^+^ T cells were analyzed separately. For gating strategy, see Supplementary Figure 3A.

In a first set of experiments, we assessed the presence of TAA-specific T cells in parous compared to nulliparous healthy females. Therefore, we collected peripheral blood samples from 30 healthy volunteers (median age: 51.5 years, mean 45.8 ± 11.7) and performed interferon γ (IFN-γ) ELISpot assays with purified CD3^+^ T cells and TAA-pulsed autologous dendritic cells (DCs) as antigen presenting cells. Donors aged 50 or older had participated in a mammography screening program and received an unsuspicious result. Primary IFN-γ ELISpot data from two exemplary donors are presented in Figure 1A. Results were defined positive (red bars), when spot numbers of test wells containing TAAs were increased at least twofold over control wells containing a negative control antigen (IgG) and the difference was statistically significant (asterisks).

We detected breast tumor-specific T cells in none of the nulliparous, but in 85% of parous women (Figure 1B). The T cell repertoire of parous women was highly polyvalent and directed against multiple different mammary epithelial-associated antigens (Figure 1C). A comparison of the total spot numbers between responding and non-responding donors verified that positive test results were not caused by low background spot numbers, but based on a significant increase in IFN-γ secretion in response to single TAAs (Supplementary Figure 1). The intensity of spontaneous anti-tumor T cell responses was not depending on the age of the volunteers, the number of full-term pregnancies, age at first delivery or previous breastfeeding (Supplementary Figures 2A-2D). These findings suggest that a single full-term pregnancy *per se* triggers a robust and long-lasting TAA-specific T cell response in women. To substantiate this assumption we were lucky to obtain blood of monozygous female triplets with distinct reproductive history. Only in the triplet who had one full-term pregnancy TAA-reactive T cells were detected, but neither in the sister with abrupt pregnancies, nor in the nulligravid one (Supplementary Figure 2E).

In order to find out at which time during or after pregnancy TAA-specific T cell responses occur, we studied blood samples of altogether 29 healthy primigravid females in gestation weeks 14 or 21, as well as within 24 hours and 4 weeks after delivery. Four volunteers donated blood at both postpartum time points. IFN-γ ELISpot analyses revealed a strong T cell reactivity against TAA already in gestation week 14 (Figure 1D), and no further increases were observed at later time points during or after pregnancy. Nor was a decrease detected in TAA-specific T cell reactivity with time elapsing after parturition. Rather, our results indicate that a breast tumor antigen-specific T cell memory can be maintained for at least 4 decades (Figure 1E).

For confirmation, we analyzed the memory phenotype of TAA-specific T cells in parous females *via* flow cytometry. For that purpose, peripheral blood T cells were stimulated with autologous dendritic cells pulsed with a mixture of four TAA or control antigen (IgG), a triple cytokine secretion assay (IFN-γ, IL-2, and TNF-α) was performed and, subsequently, the T cells were stained with fluorescently labelled antibodies against CCR7, CD45RO, CD4 and CD8 (for gating strategy, see Supplementary Figure 3). In each sample, the majority of IFN-γ-secreting TAA-specific T cells had an effector memory T cell phenotype (T_EM_, CCR7^−^ CD45RO^+^, mean: 48%), followed by lower proportions of central memory (T_CM_, CCR7^−^ CD45RO^+^), terminally differentiated effector (T_E_, CCR7^−^ CD45RO^−^), and (likely) stem cell-like memory T cells (T_SCM_, CCR7^+^ CD45RO^−^) (Figure 1F). We assume that the CCR7^+^ CD45RO^−^ cytokine-secreting population comprises T_SCM_, since naïve T cells do not secrete cytokines even 24 h after stimulation with antibody-coupled beads against CD3, CD2 and CD28 and, in our assay, antigen presentation by dendritic cells was allowed for 12 h only. Analyses detected both CD4^+^ and CD8^+^ T cells secreting IFN-γ, TNF-α and IL-2 in response to TAA. However, there was only a small proportion of polycytokine producing T cells (Figure 1G). Taken together, our results indicate that both CD4^+^ and CD8^+^ memory T cells specific for multiple breast tumor-associated antigens are induced during pregnancy and persist for decades.

### TAA-specific regulatory T cells control effector T cell responses during the breastfeeding period

**Figure 2:**
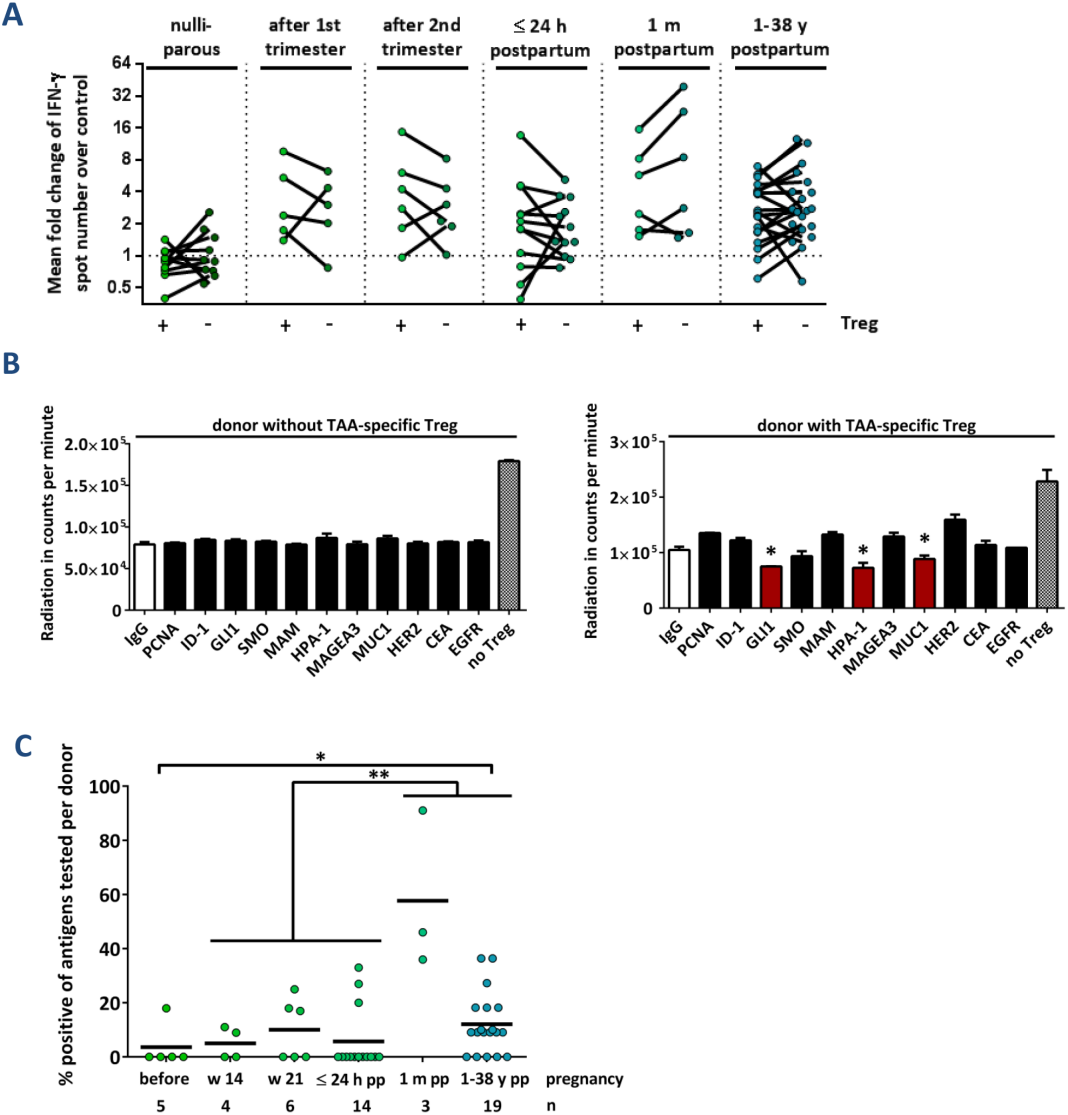
TAA-reactive T cells are suppressed *via* regulatory T cells after delivery **(A)** Cumulative results of IFN-γ ELISpot assays before (+) and after (−) depletion of CD4^+^ CD25^+^ regulatory T cells (Treg). Data points depict the mean fold change of test antigens over negative control per healthy donor with median ± interquartile range. Peripheral blood was taken from nulliparous women, from primiparous women during pregnancy at weeks 14 or 21, from primiparous women during the first 24 h after delivery, from primiparous women 1 month after delivery, and from parous women whose last pregnancy was at least 1 year before. P values were calculated using the unpaired 2-tailed t test comparing the pooled mean log fold change of test antigens over negative control. **(B)** Primary data of two exemplary regulatory T cell-specificity assays. Bars represent the mean radiation in counts per minute in 3 wells per antigen ± SEM. Suppression of proliferation by antigen-reactive Treg cells was determined by a significantly (p ≤ 0.05, unpaired t test) reduced radiation in test wells compared to the negative control IgG. ^*^ p ≤ 0.05. No Treg: Proliferation control including DCs with polyclonally activated TC without autologous Treg cells. **(C)** Cumulative results of regulatory TC-specificities in healthy individuals. Data points show the percentage of positive tests per donor. The ratio of individuals with TAA-reactive Treg cells was compared using Fisher's exact test. Cumulative data of all individuals during or instantly after pregnancy compared to donors 1 month to 38 years after childbirth ^**^ p ≤ 0.01. Cumulative data of all individuals during or instantly after pregnancy compared to donors 1 year to 38 years after childbirth ^*^ p ≤ 0.05.

To investigate whether parity-induced effector T cell responses are controlled *via* TAA-specific regulatory T cells, we performed all ELISpot assays before and after the depletion of CD4^+^ CD25^+^ regulatory T cells. As shown in Figure 2A, the depletion of Treg cells did not have an overall significant effect on the T cell responsiveness. In individual cases, however, the Treg depletion resulted in a marked increase of IFN-γ spot numbers - especially in 4 out of 6 primiparous women. Among the 4 females who donated blood 24 hours and 4 weeks after delivery, we detected strong TAA-specific T cell responses in two donors. These responses were vigorously suppressed by regulatory T cells after 4 weeks of breastfeeding, but not yet directly after delivery (Supplementary Figure 4). We thus studied the presence of TAA-specific Treg cells by a functional assay [18], which assesses the increased suppressive activity of Treg cells after antigen-specific stimulation. Primary data of two representative Treg cell-specificity experiments are presented in Figure 2B. A positive response was defined by significantly reduced Tcon proliferation after coculture with autologous TAA-stimulated Treg compared to controls with IgG-stimulated Treg cells (asterisks, red bars). Pooled results are shown in Figure 2C, representing the proportions of antigens tested positive per donor. TAA-specific regulatory T cells were detected in all groups, with the highest proportion of positive tests in primiparous women who had delivered four weeks before blood samples were taken. There was a significant increase in the number of TAAs recognized by Treg cells of primigravid compared to parous females, suggesting that more TAA-specific regulatory T cells are induced or expand after delivery. Among those healthy mothers with detectable TAA-reactive memory T lymphocytes, about one third also displayed Treg cells specific for at least one of the same antigens (Supplementary Table 1) demonstrating that T effector and Treg responses can be elicited against the same antigen. In summary, we detected TAA-specific regulatory T cells in a proportion of healthy individuals. They strictly control strong effector T cell responses during the breastfeeding period.

### TAA-specific T cell responses are improved in parous compared to nulliparous patients with breast cancer

**Figure 3:**
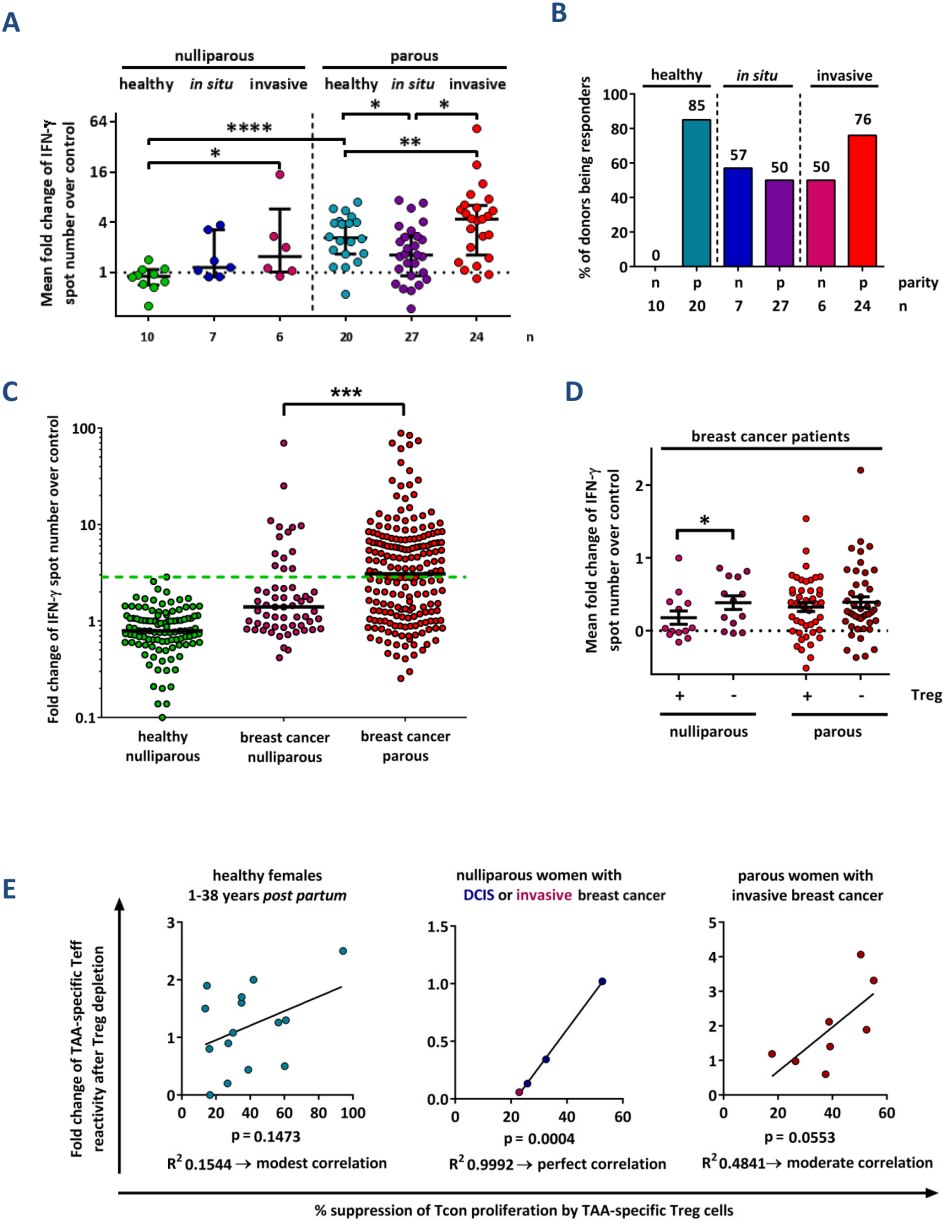
Parity improves TAA-specific T cell responses in breast cancer patients **(A)** Cumulative results of IFN-γ ELISpot analyses from healthy donors, patients with mammary ductal carcinoma *in situ* (DCIS), and patients with invasive breast cancer. Data points depict the mean fold change of test antigens over negative control per donor with median ± interquartile range. P values were calculated using the unpaired 2-tailed t test comparing the pooled mean log fold change of test antigens over negative control between healthy donors, patients with DCIS and patients with invasive carcinomas. ^*^ p ≤ 0.05, ^**^ p ≤ 0.01, ^****^ p ≤ 0.0001. **(B)** The percentage of nulliparous (n) and parous (p) females tested positive for at least one peptide in the total TC fraction is depicted. **(C)** Single test results of ELISpot assays according to the parity status of breast cancer patients. The mean change in spot number per antigen in test holes compared to the negative control (IgG) is shown. The proportion of T cell responses above the highest value measured in nulliparous healthy donors (green dashed line) was compared using Fisher's exact test. ^***^: p ≤ 0.001. Median values are shown as solid lines. **(D)** Cumulative test results of ELISpot assays according to the parity status of breast cancer patients. Data points depict the mean fold change of test antigens over negative control per healthy donor with mean ± SEM. Assays were performed before (+) and after (−) depletion of CD4+ CD25+ regulatory T cells (Treg). The significance of the effect of Treg cell depletion was calculated with the paired 2-tailed t test comparing the pooled mean log fold change of test antigens over negative control between the Treg cell-depleted and total T cell fractions obtained from the same donors. ^*^ p ≤ 0.05. **(E)** Relation of TAA-specific IFN-γ-secreting “conventional” T cells (Tcon) to TAA-specific regulatory T cells (Treg) per donor. Correlation of fold increase of IFN-γ spot number after depletion of Treg cells determined *via* ELISpot assay versus the percentage of suppression using the same antigen measured in the Treg specificity assay. Data pairs are selected from identical blood samples and include only antigens tested positive in the Treg specificity assay. Linear regression analysis was performed to calculate the goodness of fit (R^2^) and the p value.

We next investigated how TAA-specific T cell responses might be altered during breast cancer progression. Therefore, before tumor resection, we collected peripheral blood samples from 64 females (median age: 54 years; mean 55.76 ± 11.36) diagnosed with pure ductal carcinoma *in situ* (DCIS) or invasive breast cancer. The respective results of IFN-γ ELISpot assays are presented in Figures 3A-3D. T cell responses against TAA were detectable in both nulliparous women with preinvasive (*in situ*) and invasive breast cancer. In both groups, the reactivity was significantly stronger than that in nulliparous healthy females (Figures 3A, 3B). Again, the results were not biased by different background noises (Supplementary Figure 5). Regarding the antigen specificities of T cells from healthy subjects compared to breast cancer patients, we detected no major differences (Supplementary Figure 6). Median T cell responses against TAA were 2.8-fold stronger in parous compared to nulliparous patients with invasive breast cancer and we detected significantly more marked responses against single antigens (Figure 3C). However, in T cells from nulliparous breast cancer patients, the TAA responsiveness could be restored after Treg cell depletion, while T cells from parous patients were not influenced (Figure 3D). Nevertheless, TAA-specific Treg cells were frequently detected in both groups (Supplementary Figures 7-9).

Eventually, we compared the relation of TAA-specific effector to regulatory T cells in nulliparous and parous breast cancer patients. While there was a strong correlation in nulliparous breast cancer patients, it was moderate in parous breast cancer subjects and weak in healthy parous females (Figure 3E). We thus conclude that in a proportion of nulliparous women, spontaneous T cell responses against breast tumor-associated antigens are induced during breast cancer development. However, these T cell responses are more strictly controlled by Treg cells than in parous breast cancer patients.

## DISCUSSION

This study adds six important findings to the previous observation that a T cell response against a single breast cancer-associated antigen, MUC-1, is induced during the first trimester of pregnancy [16]. First, spontaneous T cell responses against TAA can be detected in most parous, but not in nulliparous women. Second, T cell reactivity is directed against a wide range of various breast cancer- associated antigens. Third, pregnancy results in the formation of a lifelong TAA-specific memory. Fourth, TAA-specific T cells have a mixed memory phenotype. Fifth, healthy females also harbor TAA-specific regulatory T cells, which control strong TAA-specific conventional T cell responses, e. g., during the breastfeeding period. Finally, in nulliparous breast cancer patients, anti-tumor T cell responses are weaker than those in parous patients, but can be enhanced *via* depletion of TAA-specific Treg cells.

Since spontaneous TAA-specific effector T cell responses correlate with a significantly improved prognosis [4–7], pregnancy-induced preexisting TAA-specific T cell responses may also protect from breast cancer development. In animal models, at least, this has already been demonstrated in that splenocytes from parous rats compared to those from nulliparous rats have revealed a significantly higher cytotoxic activity against mammary tumors *in vivo* [18]. Furthermore, the adoptive transfer of those splenocytes from parous rats into virgin counterparts resulted in a reduced carcinogen-induced mammary tumorigenesis [19]. Additional studies showed that this pregnancy-associated cytolysis was mediated by T cells in a cell contact-dependent manner.

A possible mechanism behind the induction of TAA-specific T cell responses during pregnancy is an increased expression of shared tumor- and pregnancy-associated antigens in the maturing mammary gland, fetus and/or placenta. During the first trimester of pregnancy, ductal epithelial cells of the breast undergo final differentiation and massive proliferation occurs. At this time, lymphocytes begin to accumulate in the interstitial tissue of the breast, which concurs with the induction of many breast tumor-associated antigens highly expressed in the lactating breast [13]. A previous study reported the presence of activated effector/memory T cells in breast milk, which recognized maternal epithelial surfaces [20]. Hence, there is accumulating evidence supporting the hypothesis that pregnancy implies a robust and long-lasting natural immunization against multiple tumor-associated antigens.

In this study, we demonstrated that pregnancy was also associated with an increased occurrence of TAA-reactive regulatory T (Treg) cells in approximately two thirds of healthy mothers. These often reacted against the same antigens as TAA-reactive effector/memory T cells, suggesting that peripheral tolerance might be induced to prevent autoimmune-mediated destruction of the breast. Notably, a significant increase in Treg cell responses occurred during lactation, which is in line with the observation that Treg highly infiltrate the human breast four weeks *post partum* [21].

In parous women, T cell responsiveness was not influenced by reproductive factors known to affect breast cancer risk, such as age at first childbirth or the breastfeeding history. The latter concurs with the finding that the protective impact of breastfeeding on breast cancer does not emerge until after more than 3 births [1], which applied for none of our donors. Although the lowest breast cancer risk reportedly pertains to women who had their first birth at a young age [1], we could not find superior T cell responses in mothers who had delivered early in life. Thus, assuming that the breast cancer protective effect of parity is at least partially mediated by the induction of TAA-reactive T cells, this effect might not depend on the degree of T cell responsiveness, but rather on the early onset and long duration of the immune protection.

Parity also strongly affected T cell immunity in breast cancer patients. Although we observed TAA- specific T cell responses in nulliparous patients with preinvasive and invasive breast cancer, these were weaker and more strongly suppressed by TAA-specific Treg – particularly at preinvasive stages – than those from parous breast cancer patients. We indeed detected an increased generation of TAA- reactive Treg throughout the course of breast cancer development – at least in a proportion of patients – which is in line with previous findings that Treg accumulation increases from normal tissue over ductal carcinoma *in situ* to invasive breast cancer [22].

It remains to be determined yet if the spontaneous induction of TAA-specific T cells during pregnancy eventually results in a reduced breast cancer incidence and improved prognosis in case that breast cancer still develops. Future prospective studies along these lines might provide valuable criteria to select suitable antigens for the formulation of a preventive breast cancer vaccine. Hence, since nulliparity is becoming more common in the industrialized countries and as there is a strongly increased breast cancer incidence in these women, the development and application of a preventive vaccine would be both reasonable and desirable. Clearly, to this end further studies are still needed to elucidate the mechanisms of parity-mediated breast cancer protection. Recently, for instance, alterations in Wnt and TGFβ signaling pathways in mammary stem/progenitor cells have revealed new potential targets for preventive interventions [28].

## MATERIALS AND METHODS

### Patients

Peripheral blood samples were obtained from 59 healthy donors and 64 patients with primary breast cancer without neoadjuvant therapy before primary surgery. 34 patients had a histologically approved pure ductal carcinoma *in situ* and 30 had invasive breast carcinoma. Informed consent was obtained from all participants. The study protocol was approved by the Ethical Committee of the University of Heidelberg.

### Cell culture and purification

PBMCs were collected from blood samples after Ficoll density gradient centrifugation (Biochrom, Berlin, Germany), washed and cultured in *X-VIVO* 20 medium (Lonza, Verviers, Belgium) for 1 h. Non-adherent cells were transferred to *X-VIVO* 20 medium with 100 U/ml IL-2 (Novartis, Basel, Switzerland) and 60 U/ml IL-4 (Miltenyi Biotec, Bergisch Gladbach, Germany). *X-VIVO* 20 medium supplemented with 50 ng/ml GM-CSF (Sanofi-Aventis, Frankfurt, Germany) and 1,000 U/ml IL-4 was added to adherent cells. The cells were cultivated for 5 to 10 d and subsequently deprived of cytokines for 24 h.

Dendritic cells were enriched by negative selection using Pan Mouse IgG Dynabeads® (Life Technologies, Darmstadt, Germany), coupled to mouse anti-human CD3, CD19 and CD56 antibodies, respectively. T cell purification was performed using the Untouched^™^ Human T Cells system (Life Technologies). After removal of 2.5×10^6^ T lymphocytes as non-Treg cell depleted fraction for the ELISpot assay, the remaining T lymphocytes were depleted from Treg cells using the CD4^+^ CD25^+^ Regulatory T Cell Isolation Kit (Miltenyi Biotec).

### Antigens

As TAA, we used long synthetic peptides (LSP) from seven well-characterized breast tumor-associated antigens: CEA, MAGE-A3, MUC-1, EGFR, HER2/neu, mammaglobin A, heparanase 1 and proliferating cell nuclear antigen (PCNA) and from some breast TAA without proven T cell reactivity: Inhibitor of differentiation (ID)-1 [23], glioma-associated oncogene homologue (GLI)-1 [24], and smoothened (SMO) [25]. As negative control antigen, we used human IgG. All LSP were designed to contain multiple HLA-A^*^01, HLA-A^*^0201, HLA-A^*^03 and HLA-B^*^0702 T cell epitopes, according to *in silico* prediction using SYFPEITHI, BIMAS and NetMHC 3.2, or experimentally confirmed HLA-A^*^0201 T cell epitopes, if available. As a positive control for overall assay performance, we used the bacterial superantigen enterotoxin type B (Sigma-Aldrich, St Louis, Missouri, 1 μg/ml).

The amino acid sequences of respective peptides are summarized in Table 1.

**Table 1:** Tumor antigens and synthetic long peptides

Tumor-associated antigen	Peptideposition	Peptide sequence
Proliferating cell nuclear antigen (PCNA) [27]	201–250	EPVQLTFALRYLNFFTKATPLSSTVTLSMSADVPLVVEYKIADMGHLKYN
Inhibitor of differentiation 1 (ID-1) [23]	101–146	YIRDLQLELNSESEVGTPGGRGLPVRAPLSTLNGEISALTAEAACV
Glioma-associated oncogene 1 (GLI1) [24]	365–411	KLPGCTKRYTDPSSLRKHVKTVHGPDAHVTKRHRGDGPLPRAPSIST
Smoothened (SMO) [25]	557–603	DDEPKRIKKSKMIAKAFSKRHELLQNPGQELSFSMHTVSHDGPVAGL
Mammaglobin A (Mam1) [9]	4–56	LMVLMLAALSQHCYAGSGCPLLENVISKTINPQVSKTEYKELLQEFIDDNATT
Heparanase 1 (HPA1) [11]	1–50	MLLRSKPALPPPLMLLLLGPLGPLSPGALPRPAQAQDVVDLDFFTQEPLH
MUC1 [9]	137–157	(GVTSAPDTRPAPGSTAPPAH)x5
MAGEA3 [9]	271–314	FLWGPRALVETSYVKVLHHMVKISGGPHISYPPLHEWVLREGEE
CEA [9]	569–618	YVCGIQNSVSANRSDPVTLDVLYGPDTPIISPPDSSYLSGANLNLSCHSA
HER2/neu [9]	351–384	REVRAVTSANIGEFAGCKKIFGSLAFLPESFDGD
EGFR [10]	479–528	KLFGTSGQKTKIISNRGENSCKATGQVCHALCSPEGCWGPEPRDCVSCRN

### IFN-γ ELISpot assay

The IFN-γ ELISpot assay was performed as described previously [26], with modifications.

In brief, 1×10^4^ dendritic cells were pulsed with 200 μg/ml test peptide or human IgG as negative control in triplicates. After 14 h of incubation, 5×10^4^ T cells were added. For each sample, the assay was performed with total T lymphocytes and a Treg cell-depleted fraction, respectively. After 40 h of co-culture, IFN-γ secreting T cells were visualized using an enzyme-coupled detection antibody system (Mabtech, Nacka Strand, Sweden). Plates were analyzed using an automated system (CTL, Bonn, Germany) and subjected to a manual quality control.

### Treg cell specificity assay

The Treg cell specificity assay was performed as described previously [26], with modifications.

5×10^3^ dendritic cells were pulsed with 200 μg/ml test peptide or human IgG in triplicates. After 14 h of incubation, 2.5×10^4^ CD4^+^ CD25^+^ Treg cells were added. Simultaneously, autologous Treg cell-depleted T lymphocytes (Tcon) were polyclonally activated in a plate coated with anti-human CD3 antibody. After 18 h of incubation, 2.5×10^4^ Tcon were added. After 72 h of culture, ^3^H-thymidine at 37 KBq was added. T cell proliferation was measured by determining the amount of incorporated ^3^H using the liquid scintillation counter 1450 MicroBeta (PerkinElmer, Waltham Massachusetts, USA).

### IFN-γ secretion assay

1×10^5^ DC were pulsed with 100 μg of a mixture of polypeptides (ID-1, mammaglobin-A, MUC1, and heparanase 1) or human IgG. 8 h later, 5×10^5^ TC were added. After 12 h of coincubation, unspecific binding to Fc receptors was blocked by preincubation of cells for 20 minutes with human IgG (Sandoglobin, Sigma-Aldrich, Taufkirchen, Germany). Secreted IFN-γ was captured and fluorescently labelled using the MACS human IFN-γ Secretion Assay Detection Kit (Miltenyi), according to the manufacturer's instructions. Subsequently, TC were stained for CD4, CD8, CD45RO, CCR7 (clones RPA-T4, RPA-T8, UCHL1 and 3D12, respectively, all from BD Biosciences, Heidelberg, Germany) and viability (LIVE/DEAD Fixable Yellow Dead Cell Stain, BD). Isotype control stainings were performed using corresponding isotype antibodies. Samples were analyzed using a FACS Canto II flow cytometer (BD) and FlowJo software (vX, TreeStar, Ashland, USA).

### Statistical evaluation

T cell reactivity in ELISpot experiments was calculated as the fold increase of spot numbers in test wells over that in negative control wells, as a mean of all tests per patient. A response was defined positive when test wells had at least twofold higher spot counts than the negative control wells and the difference was significant in the unpaired 2-tailed t test. Cumulative ELISpot data were analyzed as pooled mean log fold change of test antigens over negative control. The unpaired 2-tailed t test was used to compare different groups of donors. The effect of Treg cell-depletion within the same group was assessed by the paired 2-tailed t test.

The presence of TAA-specific Treg cells was defined positive when test peptide wells had significantly lower scintillation counts than negative control wells, as determined by unpaired 2-tailed t test. Proportions of responders were compared between groups using the Fisher's exact test. Differences were considered statistically significant when p ≤ 0.05.

## SUPPLEMENTARY MATERIALS FIGURES AND TABLE


