# Supplementary material for: Parity improves anti-tumor immunity in breast cancer patients
Source: Oncotarget. 2017 Sep 8;8(62):104981–91. doi: 10.18632/oncotarget.20756 (PMC5739613; doi:10.18632/oncotarget.20756)
Supplement: Supplementary file 1 [file oncotarget-08-104981-s001.pdf]

Parity improves anti-tumor immunity in breast cancer patients

SUPPLEMENTARY MATERIALS

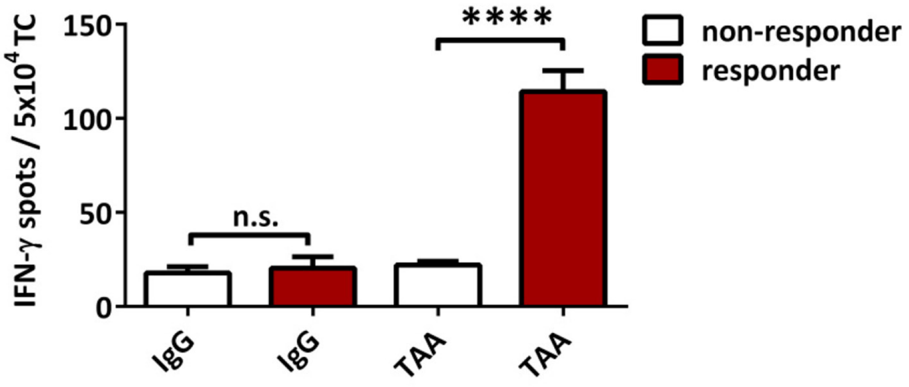

Supplementary Figure 1: Cumulative results of the IFN- γ ELISpot assay with peripheral blood T cells from healthy donors. Total mean IFN-γ spot numbers for control antigen (IgG) in responding (red) and non-responding (white) healthy donors, and for test antigens tested positive (red) or negative (white), respectively. Bars: mean ± SEM. TC: T cells. **** $p \leq 0.0001$ (unpaired 2-tailed t test). IgG: n=13-17; TAA: n=91-239.

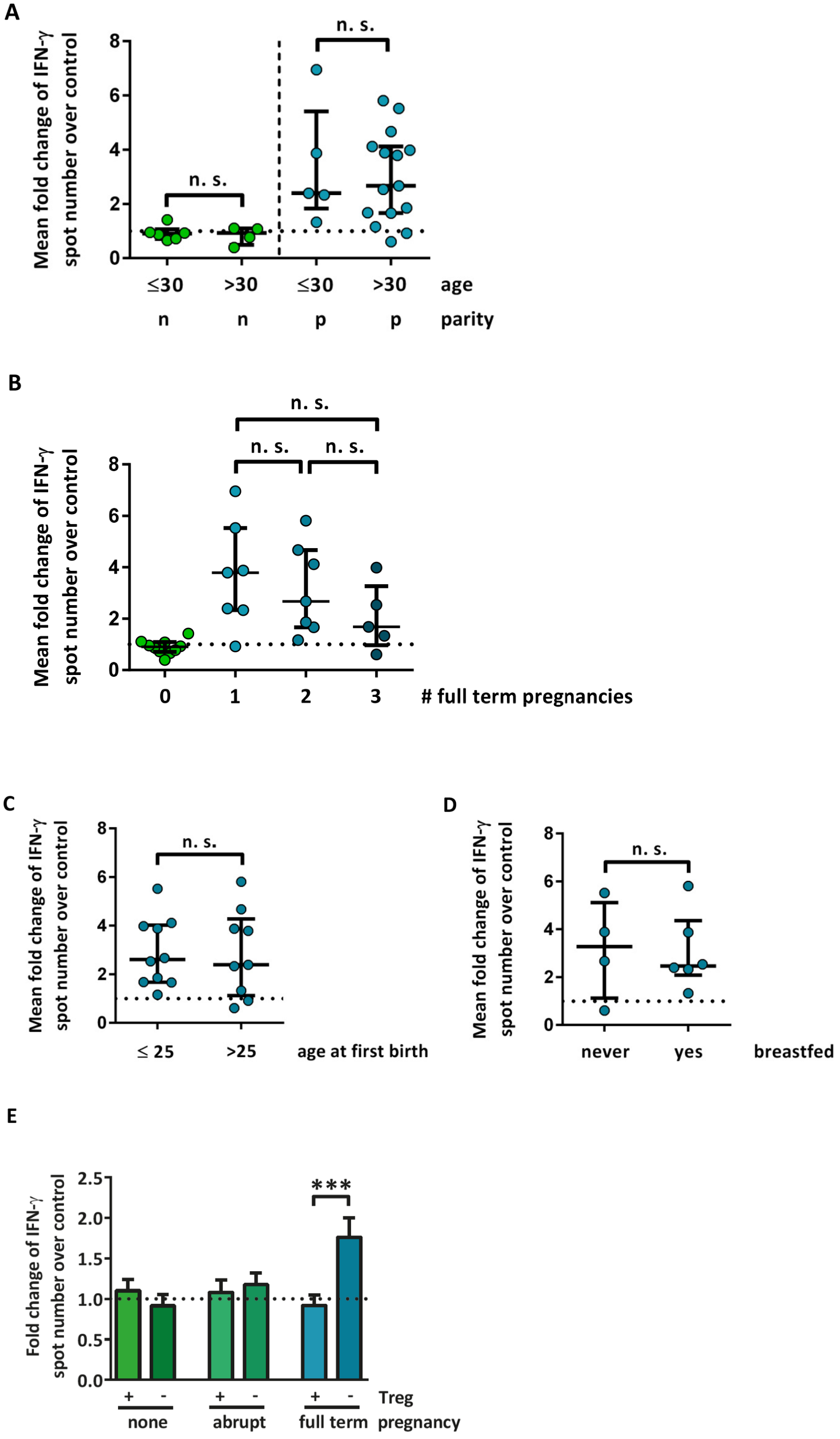

Supplementary Figure 2: Cumulative results of IFN-γ ELISpot assays separated according to **(A)** age, **(B)** number of full-term pregnancies, **(C)** age at first birth, and breastfeeding history **(D)** of healthy donors. Data points depict the mean fold change of test antigens over negative control per donor with median ± interquartile range. **(E)** Exemplary IFN-γ ELISpot results from peripheral blood of female monozygous triplets. The first triplet is nulligravida, the second had two aborted ectopic pregnancies and the third had one full-term pregnancy. Bars represent the fold change of test antigens over the negative control ± SEM. *** $p \leq 0.001$ (paired 2-tailed t test). Treg (+) and (-): before and after depletion of $CD4^+$ $CD25^+$ Treg cells.

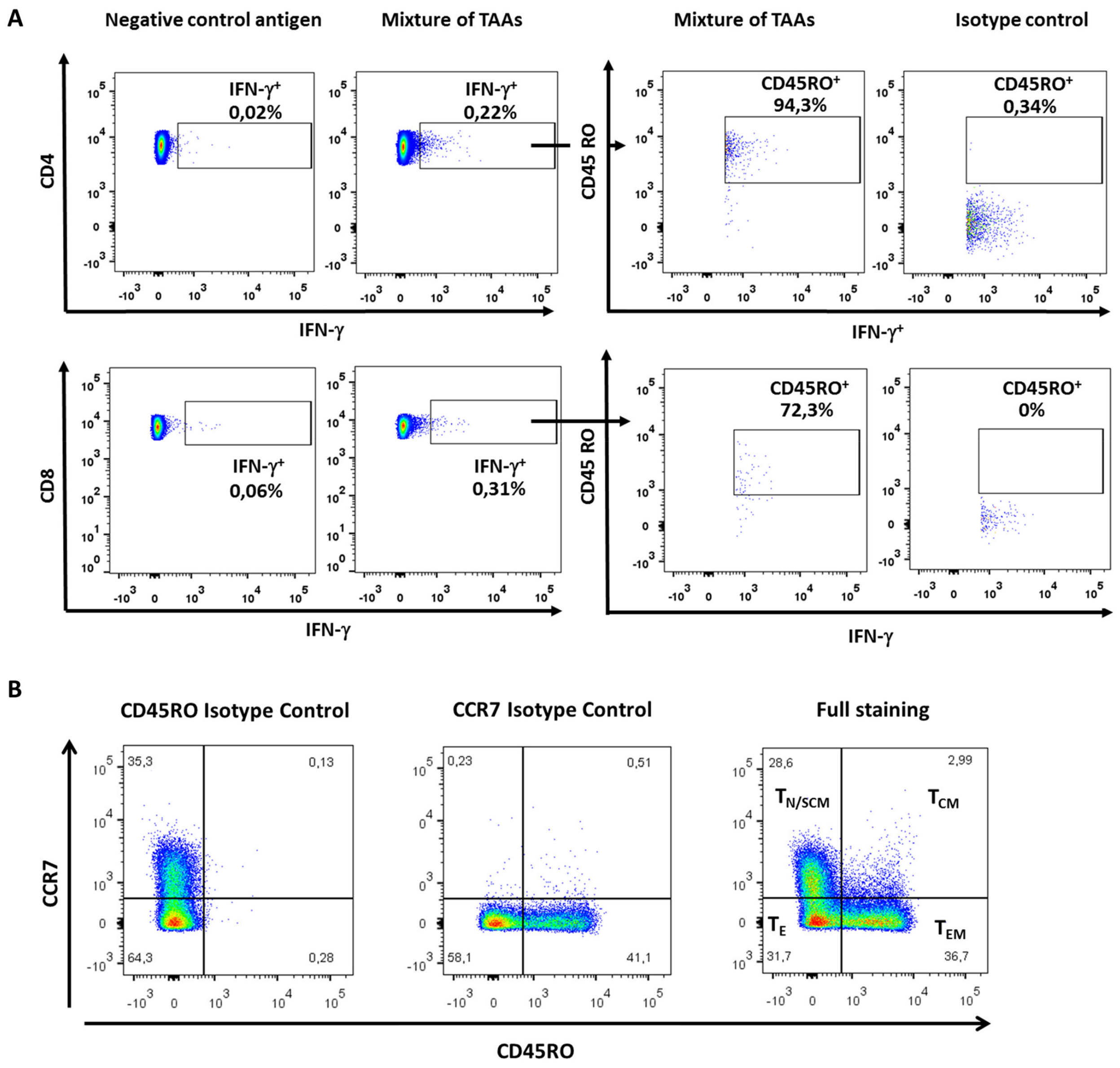

Supplementary Figure 3: Flow cytometric analysis of TAA-specific **(A)** and total **(B)** T cells after performance of a cytokine secretion assay. (A) Left panel: Gates show percentage of IFN-γ-secreting $CD4^+$ or $CD8^+$ T cells after stimulation with human IgG or peptide mix including MUC1, mammaglobin A, ID-1, and heparanase 1. Right panel: CD45RO expression on TAA-stimulated IFN-γ^+ T cells and respective isotype controls. (B) Gating strategy for discrimination of naïve/stem cell-like memory T cells: (T_N/T_{SCM}, $CCR7^+$ $CD45RO^-$), central memory T cells (TCM, $CCR7^+$ $CD45RO^+$), effector memory T cells (T_{EM}, $CCR7^-$ $CD45RO^+$), and terminally differentiated effector T cells (T_E, $CCR7^-$ $CD45RO^-$).

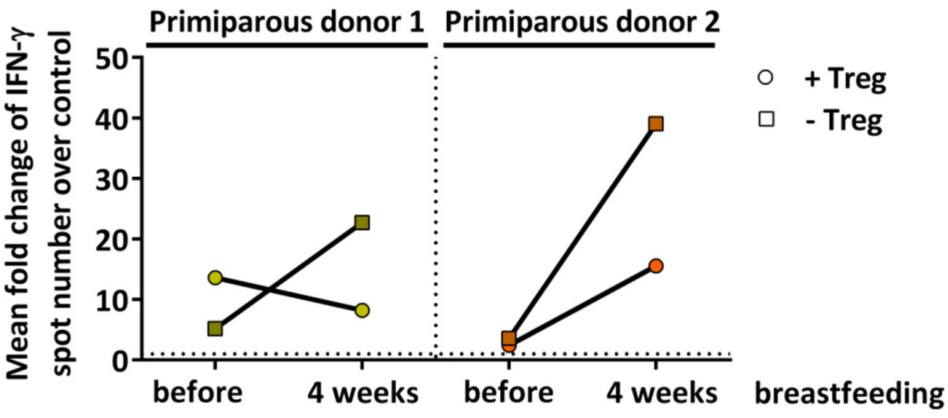

Supplementary Figure 4: Exemplary IFN-γ ELISpot results obtained from two primiparous healthy donors who donated peripheral blood ≥ 24 h and 1 month after pregnancy. Both donors had been breastfeeding until the second blood donation. Data points indicate the mean fold change of test antigens over negative control before (+) and after (-) depletion of $CD4^+$ $CD25^+$ Treg cells.

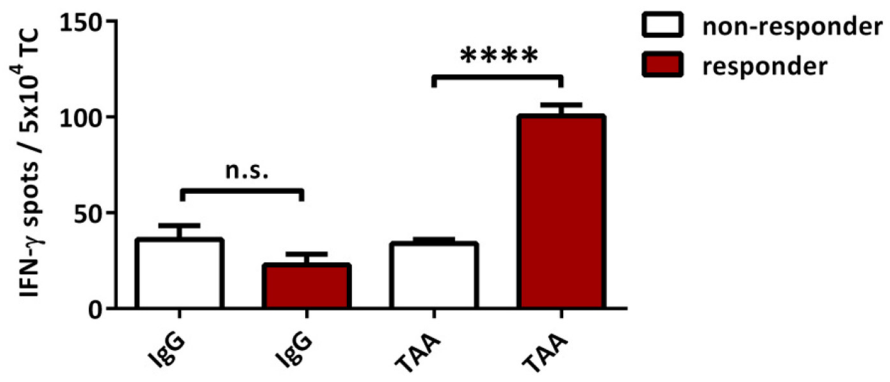

Supplementary Figure 5: Cumulative total mean IFN-γ spot numbers from Treg cell-undepleted T cells for control antigen (IgG) in responding (red) and non-responding (white) healthy donors, as well as for test antigens (TAA) tested positive (red) or negative (white). Mean ± SEM. TC: T cells. **** $p \leq 0.0001$. IgG: n=43–78; TAA: n=342-860.

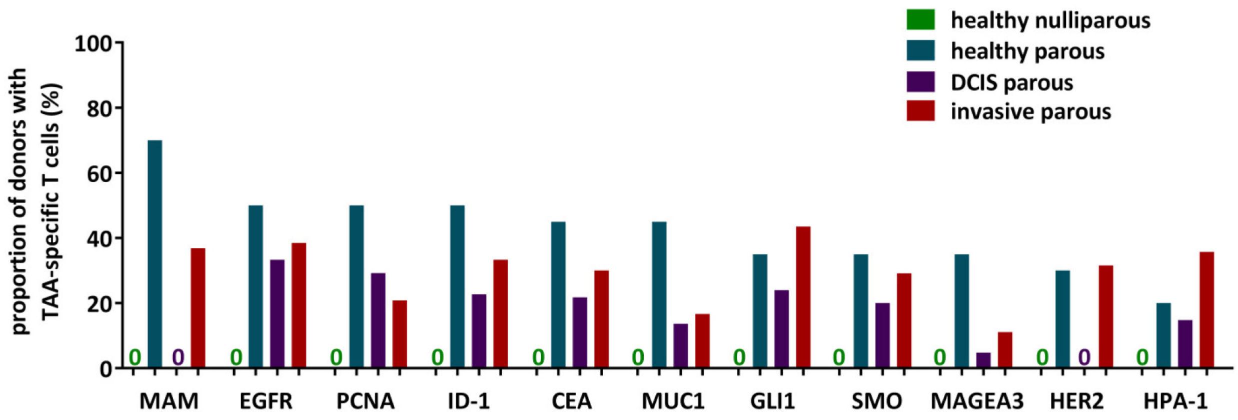

Supplementary Figure 6: Percentages of healthy donors and breast cancer patients with T cells reactive against the respective antigen. Cumulative results of IFN-γ ELISpot assays. N=6-27 individuals per antigen. 0: not detected

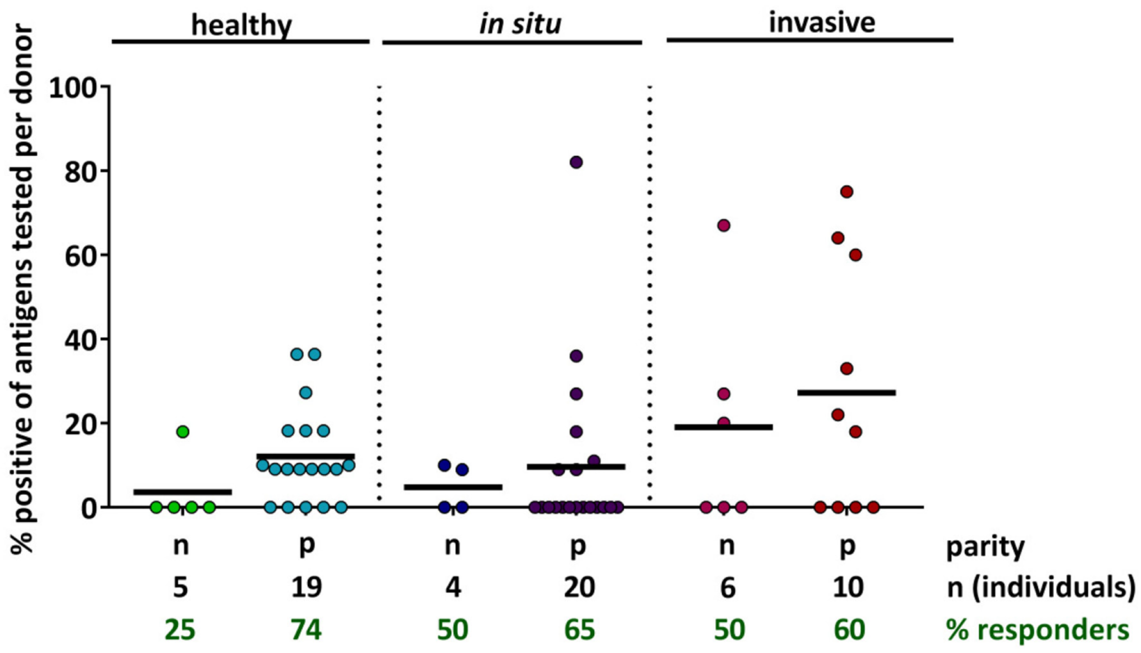

Supplementary Figure 7: Cumulative results of the Treg cell-specificity assay. Data points represent the number of tested antigens that were recognized by Treg cells of one individual. N=4-11 tested antigens per donor. Horizontal lines display the mean value (solely for illustration).

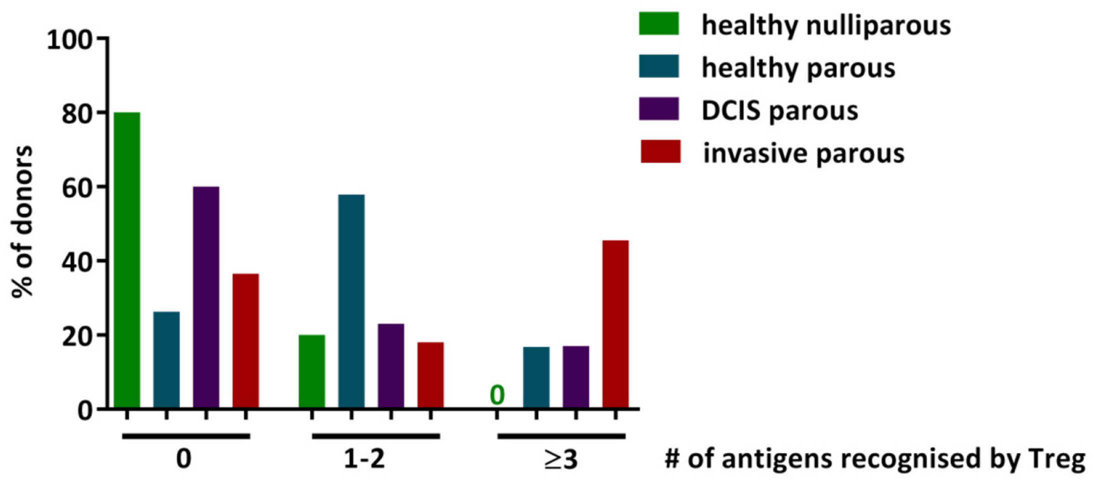

Supplementary Figure 8: Proportion of healthy donors and breast cancer patients with no (0), 1-2, or at least 3 positive tests in the Treg cell-specificity assay. At least 4 antigens were tested per individual.

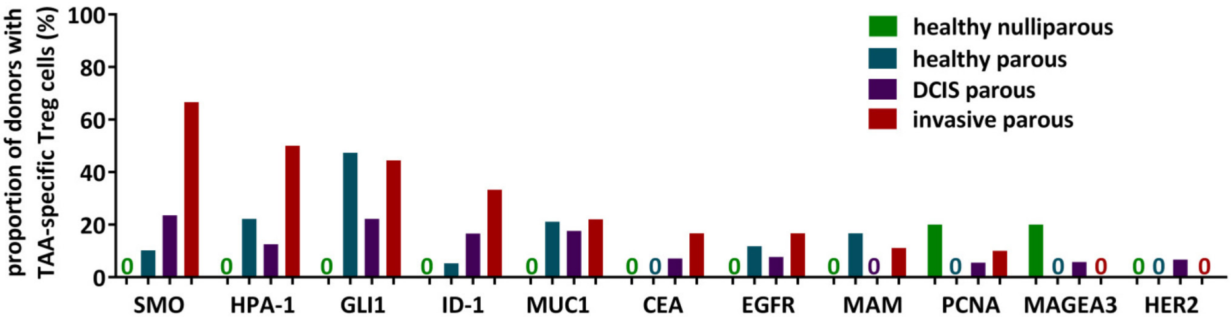

Supplementary Figure 9: TAA-specificities of Treg cells. Data represent the percentage of individuals with Treg cells reactive against the respective antigen. Results from the Treg cell-specificity assay. N=5-21 individuals per antigen. 0: not detected.

Supplementary Table 1: Tumor antigens recognized by memory and Treg cells from healthy parous women

Donor	Tmem	Treg
1	PCNA, ID-1, Gli1, Mam, HPA, EGFR	*not done*
2	ID-1, Gli1, Smo, CEA, EGFR	*not done*
3	Gli1, HPA, Her2, CEA, EGFR	*not done*
4	EGFR	Muc1
5	PCNA, ID-1, Gli1, Smo, Mam, HPA, MAGE, Her, EGFR	-
6	-	-
7	PCNA, ID-1, Gli1, HPA, Muc, Her, CEA	Mam
8	PCNA, ID-1, Smo, Mam, HPA, Muc, EGFR	Gli1, HPA, Muc
9	PCNA, ID-1, Gli1, Smo, Mam, HPA, Muc1, Her, CEA, EGFR	Gli, Smo, HPA, Muc
10	PCNA, HPA-1, MAGE, Her2	Gli
11	-	Gli, EGFR
12	PCNA, ID-1, Smo, Mam, HPA, MAGE, Muc1, Her, CEA, EGFR	Gli
13	ID-1, Gli, Smo, Mam, HPA, Muc1, Her, CEA, EGFR	Gli
14	HPA1	Mam
15	PCNA, HPA, MAGE, Her	-
16	PCNA, HPA, Muc1	HPA
17	PCNA, ID-1, Gli1, Smo, Mam, HPA, Muc1	EGFR
18	HPA-1, Her2	-
19	ID-1, EGFR	-
20	-	*not done*
